# Endometriosis is a disease of immune dysfunction, which could be linked to microbiota

**DOI:** 10.3389/fgene.2024.1386411

**Published:** 2024-06-21

**Authors:** Hongyan Liu, Junxia Li, Chenchen Guan, Wenjie Gao, Yan Li, Jianmei Wang, Yang Yang, Yongrui Du

**Affiliations:** ^1^ Department of Family Planning, The Second Hospital of Tianjin Medical University, The Province and Ministry Co-sponsored Collaborative Innovation Center for Medical Epigenetics, Tianjin Key Laboratory of Inflammatory Biology, School of Basic Medical Sciences, Tianjin Medical University, Tianjin, China; ^2^ Department of Bioinformatics, School of Basic Medical Sciences, Tianjin Medical University, Tianjin, China

**Keywords:** gut microbiota, endometriosis, GWAS, two-sample Mendelian randomization, FinnGen

## Abstract

**Background:** Endometriosis, characterized by extrauterine endometrial tissue, leads to irregular bleeding and pelvic pain. Menstrual retrograde theory suggests fragments traverse fallopian tubes, causing inflammation and scar tissue. Prevalent among infertile women, risk factors include fewer pregnancies, delayed childbirth, irregular cycles, and familial predisposition. Treatments, medication, and surgery entail side effects. Studies link gut microbiota alterations to endometriosis, necessitating research to establish causation. We used Mendelian randomization to investigate the potential link between endometriosis and gut microbiota through genetic variants.

**Methods:** Two-sample Mendelian randomization analyzed gut microbiota’s potential causal effects on endometriosis. Instrumental variables, robustly associated with exposures, leveraged GWAS data from MiBioGen for gut microbiota and FinnGen R8 release for endometriosis. SNPs strongly associated with exposures were instrumental variables. Rigorous assessments ensured SNP impact scrutiny on endometriosis.

**Results:** At the genus level, *Anaerotruncus*, *Desulfovibrio*, *Haemophilus*, and *Holdemania* showed causal association with endometriosis. Specific gut microbiota exhibited causal effects on different endometriosis stages. *Holdemania* and Ruminococcaceae *UCG002* exerted reversible, stage-specific impacts.

**Conclusion:** Mendelian randomization provides evidence for the causal link between specific gut microbiotas and endometriosis, emphasizing the pivotal role of gut microbiota dysbiosis. Modulating gut microbiota emerges as a promising strategy for preventing and treating endometriosis.

## Introduction

Endometriosis, defined as the presence of endometrial tissue outside the uterine cavity, typically lines various locations such as the ovaries, fallopian tubes, vagina, or other parts of the uterus (Vercellini et al., 2014), manifests with ectopic endometrial tissue that develops and detaches during the menstrual cycle, leading irregular bleeding. While the prevalence of endometriosis in fertile women ranges from 10% to 15%, the incidence rate significantly rises to 20%–50% in cases of infertility (Tanbo and Fedorcsak, 2017; Zondervan et al., 2020). The widely accepted theory of menstrual retrograde posits, proposed by Sampson (Sampson, 2024), that endometrial fragments retrograde through the fallopian tube, contributing significantly to the etiology of endometriosis. This process can induce inflammation and scar tissue formation, resulting in pelvic pain, sexual discomfort, menstrual irregularities, and even infertility (Tanbo and Fedorcsak, 2017; Peiris et al., 2018). Research has revealed differences in both the quantity and activation status of immune cells within the endometrium between patients with endometriosis and normal females (Agic et al., 2006; Saraswat et al., 2017; Patel et al., 2018; Vallvé-Juanico et al., 2019; Bunis et al., 2022). The aberrant expression of these immune cells may contribute to the development and progression of endometriosis, exerting adverse effects on embryo implantation and reproductive outcomes (Saraswat et al., 2017). Additionally, the ectopic endometrium of endometriosis patients harbors a spectrum of immune cells associated with both innate and adaptive immune systems, collectively creating a conducive environment for the ectopic endometrial growth (Vallvé-Juanico et al., 2019; Bunis et al., 2022).

The symbiotic association between the host and microbiota is characterized by mutual benefits (Shi et al., 2017). The host serves as a vital habitat, supplying essential nutrients to sustain the microbiome, while the gut microbiota, in turn, aid in the development of the metabolic system and facilitate the maturation of the immune system through the provision of advantageous nutrients (Kau et al., 2011). Nevertheless, alterations in the equilibrium of gut microbiota communities can lead to dysbiosis, triggering diseases (Rooks and Garrett, 2016; Shi et al., 2017). Numerous studies have underscored the correlation between alterations in gut microbiota and diverse disorders, emphasizing their impact on systemic inflammation and immune cell function (Karmarkar and Rock, 2013; Costello et al., 2015). Certain studies have specifically explored the correlation between the intestinal flora and the etiology of endometriosis (Chadchan et al., 2021; Salliss et al., 2021). Particularly noteworthy is the study by Ata et al., which identified elevated levels of Gardnerella, *Streptococcus*, *Escherichia*, *Shigella*, and Ureoplasma in women with endometriosis (Ata et al., 2019). Additionally, Acidovorax, Devosia, Methylobacterium, Phascolarctobacterium, and *Streptococcus* abundance in the peritoneal fluid of endometriosis patients surpassed that in controls (Yuan et al., 2022). The imbalance of gut microbiota can lead to an imbalance of the immune system, resulting in a dysregulation of immune cells and their corresponding pathways. While the microbiota composition in the vaginal, cervical, and intestinal regions exhibits similarities between ASRM (American Society for Reproductive Medicine) stage 3/4 endometriosis patients and controls, variations at the genus level are evident (Author Anonymous, 1997; Ata et al., 2019). They substantiated an association between gut microbiota, serum hormones, and inflammatory factors in endometriosis (Shan et al., 2021). Current primary treatment methods for endometriosis include medication and surgery. Hormonal therapies, such as GnRH agonists and Dienogest, are commonly employed to manage symptoms but come with side effects like mood instability, perimenopausal symptoms, and decreased bone density (Vercellini et al., 2014). Surgery, often utilized to remove endometriosis lesions and scar tissue, is associated with a high recurrence rate (Shakiba et al., 2008; Guo, 2009; Horne and Missmer, 2022). Frequent surgeries not only pose a risk to surrounding organs but also impose substantial economic and psychological burdens on patients. In addition, Zizolfi et al. have provided a comprehensive review of the current research on the interplay between microbiota and endometriosis, and suggested potential therapeutic interventions, including antibiotics, probiotics, and prebiotics, as well as novel approaches such as fecal, vaginal, or uterine microbial transplantation, to restore a dysbiotic state to a more favorable genital microenvironment (Zizolfi et al., 2023).

In this study, we explored the potential relationship between the microbiota and endometriosis and emphasizing this perspective as a focal point in future research to uncover underlying mechanisms, holds promise for paving the way towards the development of new approaches in preventing and treating endometriosis. This would bring hope to patients who bear the burden of endometriosis.

Mendelian randomization (MR), a novel statistical approach utilizing genetic variants as instrumental variables, provides a robust method to explore the causal link between exposure and outcome. This study employed Mendelian randomization analysis to explore the connection between endometriosis and gut microbiota. The analysis considered nine phyla, 16 classes, 20 orders, 32 families, and 119 genera (excluding 3 unknown families and 12 unknown genera). Endometriosis GWAS summary statistics from the FinnGen consortium R8 release data of European ancestry and gut microbiota GWAS summary statistics from the MiBioGen collaboration, predominantly of European ancestry, were collected for this purpose.

## Materials and methods

### Study design

**FIGURE 1:**
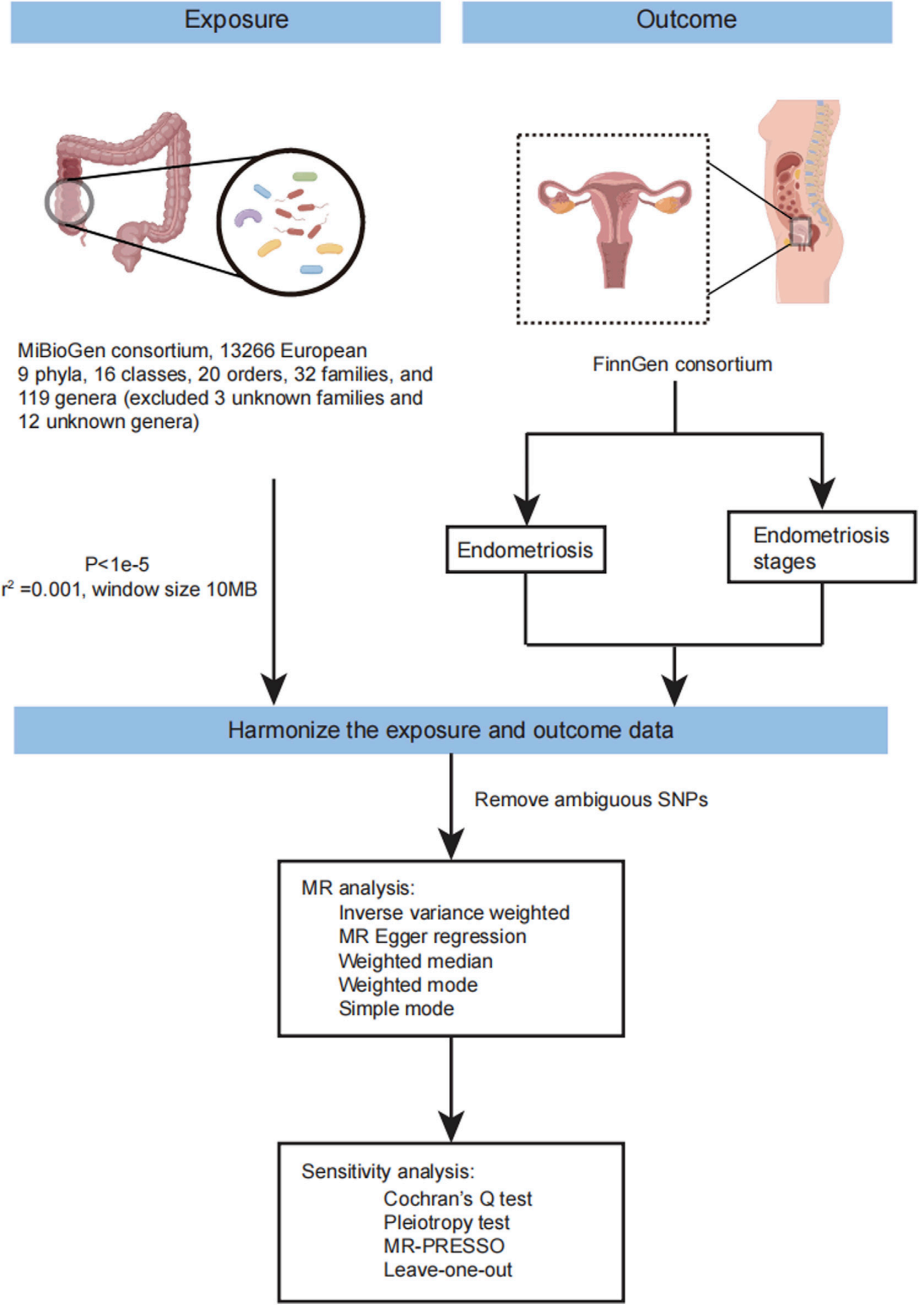
The workflow of MR analysis in this study. The flow chart showing the step of MR analysis in this study.

Three prerequisites must be met in Mendelian randomization (MR) analysis: 1) instrumental variables should exhibit an association with the exposure; 2) instrumental variables should not be correlated with confounders; and 3) genetic variants must solely influence the outcome through the exposure. The flow of MR analysis is illustrated in Figure 1. Leveraging publicly available GWAS data on relevant gut microbiota, we explored the causal association between endometriosis and gut microbiota. For each taxonomic group, an instrumental variable was selected based on summary statistics. Summary-level data for endometriosis were sourced from FinnGen. The genus was the lowest taxonomic level in the study of Kurilshikov A et al., so we mainly analyzed the causal effects between the genus level of gut microbiota and endometriosis. Endometriosis is classified into four stages by the American Society of Reproductive Medicine (ASRM) based on the disease’s location, extent, and depth within pelvic structures (Author Anonymous, 1997; Zondervan et al., 2020). Stage I involves superficial lesions, Stage II includes both superficial and some deep lesions, Stage III comprises lesions with adhesions between the ovaries and fallopian tubes, and Stage IV is characterized by severe adhesions in the pelvic region and damage to the pouch of Douglas. Subsequently, MR analysis was conducted to estimate the causal effects of the genus on different stages of endometriosis.

### Data sources

Genetic variants of gut microbiota were obtained from the large-scale association analyses by MiBioGen consortium (Kurilshikov et al., 2021). The data was collected from 24 cohorts, including 18,340 individuals, most of whom had European ancestry (13,266 individuals). This study included nine phyla, 16 classes, 20 orders, 32 families, and 119 genera for analysis (we excluded the 3 unknown families and 12 unknown genera). In this study, the genus was the main analysis object at the lowest taxonomic level. GWAS summary statistics for endometriosis were obtained from FinnGen consortium R8 release data (Kurki et al., 2023). The GWAS summary statistics data of endometriosis included 13,456 cases. Endometriosis was defined by E80 in ICD-10 and 617 in ICD-9, and 6253 in ICD-8. In addition, we also acquired the GWAS summary statistic data in different stages of endometriosis based on ASRM. FinnGen combined stages 1 and 2 (5122 cases, 185,757 controls) and merged stages 3 and 4 (6751 cases, 184,128 controls).

### Selected instrumental variables

In this MR analysis, the genus of gut microbiota was divided into 119 taxa (we eliminated the 12 unknown genera). (https://mibiogen.gcc.rug.nl/). We selected genetic variants significantly associated with gut microbiota using a threshold of *P* < 1e-5, which is consistent with previous studies (Li et al., 2022; Liu et al., 2022; Yu et al., 2023) as instrumental variables for further MR analysis (Supplementary Material S1; Supplementary Table S1). We also considered the linkage disequilibrium (LD) between SNPs. SNPs with high LD may have correlated effects and may inflate the statistical significance of the MR analysis. Then we remove SNPs in strong linkage disequilibrium (LD) which $r^{2}>0.001$ , which is consistent with previous studies (Li et al., 2022; Liu et al., 2022; Yu et al., 2023), and clumping distance<10 MB using the European reference panel of the 1000 Genomes projects to ensure the instrumental variables for each exposure are independent. Finally, we calculated *F*-statistic to estimate the strength of instrumental variables to satisfy the MR assumption by following the equation $F=\frac{\beta^{2}}{{SE}^{2}}$ (Bowden et al., 2016). SNPs with F-statistics greater than 10 were retained as instrumental variables for further analysis in our study.

### Statistical analysis

Harmonizing Single-Nucleotide Polymorphism effects is crucial in MR analyses to ensure that the effect alleles are the same for both exposures and outcomes. Common sources of bias include wrong-effect alleles, palindromic SNPs, and incompatible alleles. Palindromic SNPs pose challenges in identifying the effect allele and should be excluded. This study used various methods, including the random-effects inverse variance weighted method, MR-Egger regression, weighted median (WM), Simple mode, and weighted mode. The primary analysis used the inverse variance weighted method, which meta-analyses the Wald estimates for each SNP to evaluate the association. To acquire a robust result, we also performed MR-PRESSO. Additionally, we conducted MR analyses based on the summary statistics data of different stages of endometriosis from FinnGen.

MR-Egger regression is a method based on the inverse variance weighted method that satisfies the assumptions of instrument strength independent of direct effect (InSIDE) and no measurement error (NOME) (Bowden et al., 2015; Spiller et al., 2019). Compared to the IVW method, the MR-Egger regression method includes an intercept, which helps determine the presence of pleiotropy between instrumental variables and outcome. Therefore, we used MR-Egger regression to detect horizontal pleiotropy. Based on Cochran’s Q test, heterogeneity was evaluated using MR-Egger regression and IVW. MR-PRESSO detected and corrected horizontal pleiotropy by removing outliers in the casual relationship (Verbanck et al., 2018). We used MR-PRESSO global test to detect pleiotropy, and if significant (*p* < 0.05), outliers identified by the MR-PRESSO outlier test were removed, and the MR analysis was repeated. Finally, we used leave-one-out analysis to assess whether the impact of a single SNP affects the results and has the horizontal pleiotropic effect. We eliminate SNP individually and recalculate the MR analysis to identify which SNP is causing change (Emdin et al., 2017).

To avoid multiple comparison problems, we use the FDR method to adjust *p*-value in this study. MR-PRESSO global test and outlier test were implemented in R package MR-PRESSO. All analyses were performed by TwoSampleMR (v 0.5.6) package in R software (version 4.0.3) (R Core Team (2020), R: A language and environment for statistical computing. R Foundation for Statistical Computing, Vienna, Austria. URL https://www.R-project.org/).

## Results

### Selection of instrumental variables

We selected genome-wide significant SNPs association with gut microbiota from MiBioGen consortium. These gut microbiotas were divided into five levels: phylum, class, order, family, and genus. We set the *p*-value threshold at 1e-5, consistent with previous studies (Li et al., 2022; Liu et al., 2022; Yu et al., 2023). Then, we remove the single nucleotide polymorphisms with a linkage disequilibrium (LD). To keep the direction of effect allele of exposure and outcome are same, ambiguous SNPs with non-concordant alleles and palindromic SNPs with ambiguous strands that cannot be corrected were discarded in the harmonizing step. So, the SNPs we used may be equal to or less than that listed in Supplementary Material S1; Supplementary Table S1. We calculated the F statistic for each SNP and remained the SNPs with the F-statistic greater than 10 for the following analysis. (Supplementary Material S1; Supplementary Table S1).

### A causal association between gut microbiota and endometriosis

We analyzed MR to explore the causal association between gut microbiota and endometriosis. The MR estimates used different methods, shown in Supplementary Material S1; Supplementary Tables S2–S7. In order level of gut microbiota, the *Burkholderiales* (IVW: OR = 1.21, 95%CI: 1.02–1.42, *p* = 0.027) and *Rhodospirillales* (IVW: OR = 0.90, 95%CI: 0.82–0.99, *p* = 0.029; WM: OR = 0.87, 95%CI: 0.76–0.99, *p* = 0.039) have a causal relationship with endometriosis at the order level of the gut microbiota. (Supplementary Material S1; Supplementary Table S4) We performed the MR-Egger intercept test and Cochran’s Q test; we did not observe evident horizontal pleiotropy and heterogeneity and no potential, influential instrumental variable in the leave-one-out analysis for *Burkholderiales* and *Rhodospirillales.* In brief, *Burkholderiales* is a risk factor in endometriosis progression, it can increase the risk of endometriosis, and *Rhodospirillales* can decrease the risk of endometriosis; it is a protecting factor in the development of endometriosis.

In family level of gut microbiota, *Clostridialesvadin BB60 group* decreased the risk of endometriosis (IVW: OR = 0.86, 95%CI: 0.78–0.95, *p* = 0.003; WM: OR = 0.86, 95%CI: 0.75–0.99, *p* = 0.041). Oxalobacteraceae can reduce the risk of endometriosis (IVW: OR = 0.91, 95%CI: 0.85–0.98, *p* = 0.014). Porphyromonadaceae increases the risk of endometriosis (IVW: OR = 1.27, 95%CI: 1.03–1.56, *p* = 0.027) and lower by Rhodospirillaceae (IVW: OR = 0.91, 95%CI: 0.83–1.00, *p* = 0.048). (Supplementary Material S1: Supplementary Table S5) *Clostridialesvadin BB60 group,* Oxalobacteraceae*,* Porphyromonadaceae*,* and Rhodospirillaceae had no horizontal pleiotropy and heterogeneity. In a word, Porphyromonadaceae is a risk factor for endometriosis; it can increase the risk or aggravate endometriosis. The rest of these significant gut microbiota are protecting factors in the development of endometriosis.

**FIGURE 2:**
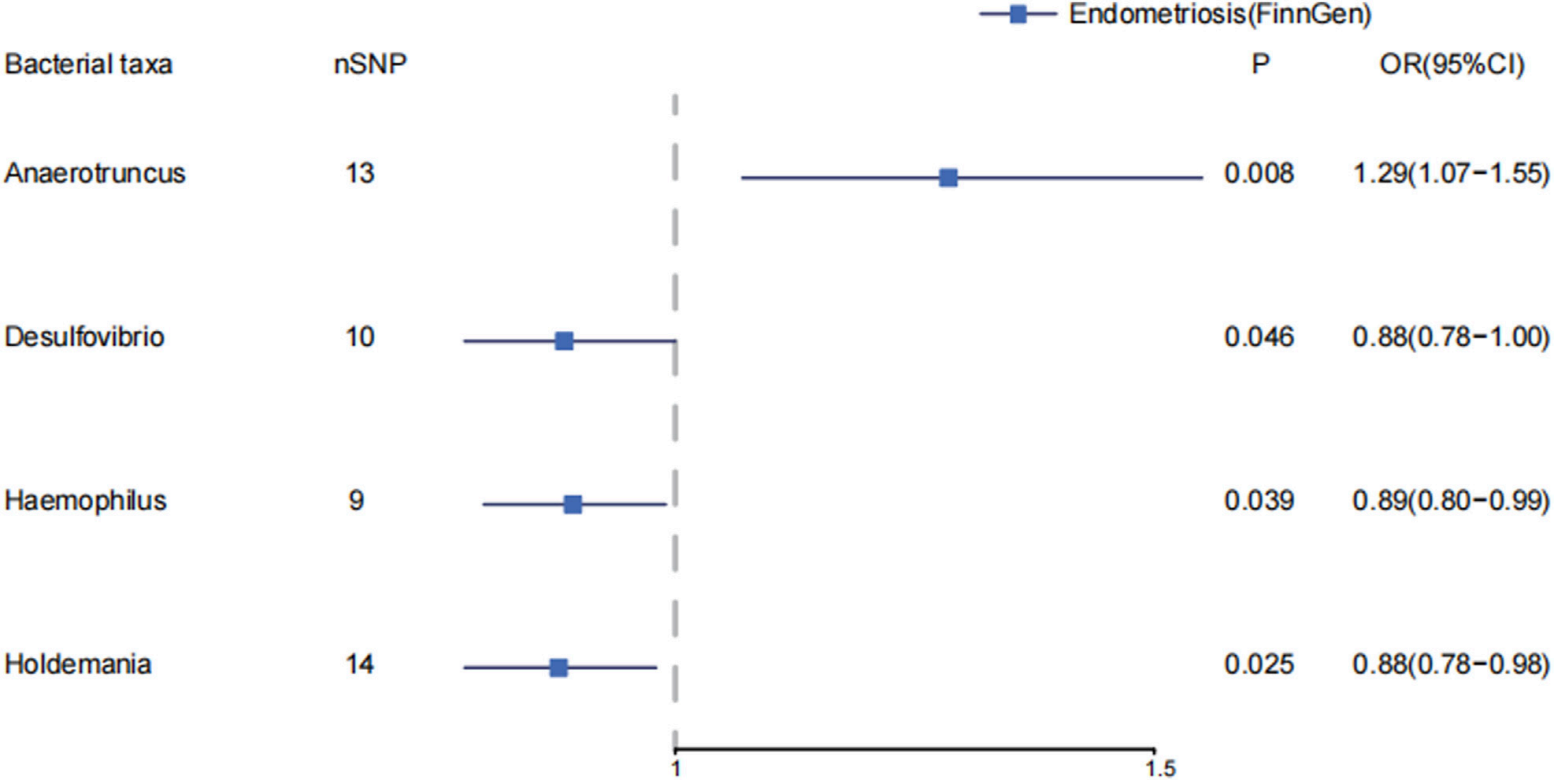
The causal effects of the genus level of gut microbiota on endometriosis in FinnGen by MR analysis. Forest plot depicting the significant causal effects of the genus level of gut microbiota on endometriosis in FinnGen. The odds ratio (OR) was estimated using IVW method. The horizontal bars represent 95% confidence intervals (CI).

In genus level of gut microbiota, *Anaerotruncus* increase the risk of endometriosis (IVW: OR = 1.29, 95%CI: 1.07–1.55, *p* = 8.31E-3; WM: OR = 1.22, 95%CI: 1.00, *p* = 0.047). In MR-PRESSO, *Anaerotruncus* still have a causal association with endometriosis (*p* = 0.013). (Supplementary Material S1; Supplementary Tables S6–S7) (Figure 2) *Desulfovibrio* decreases the risk of endometriosis (IVW: OR = 0.88, 95%CI: 0.78–1.00, *p* = 0.046). *Haemophilus* decrease the risk of endometriosis (IVW: OR = 0.89, 95%CI: 0.80–0.99, *p* = 0.039). *Holdemania* decrease the risk of endometriosis (IVW: OR = 0.88, 95%CI: 0.78–0.98, *p* = 0.025).

### The casual association between genus of gut microbiota and minimal to mild endometriosis

**FIGURE 3:**
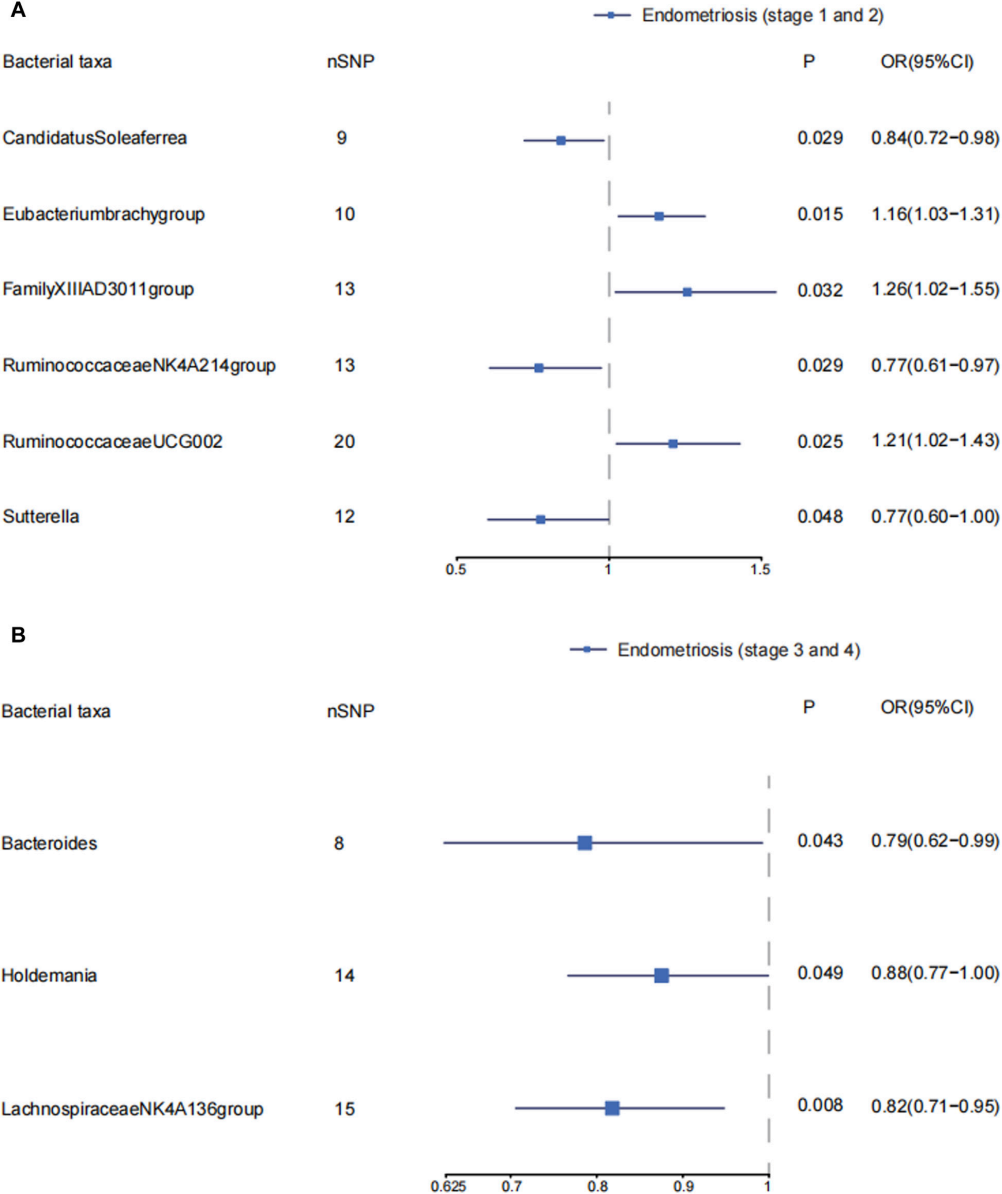
The causal effects of the genus level of gut microbiota on different endometriosis stages. Forest plots depicting causal estimates of the genus level of gut microbiota on different stages of endometriosis in FinnGen. **(A)** The significant causal association between genus level of gut microbiota and stage 1 and 2 of endometriosis. **(B)** The significant causal association between genus level of gut microbiota and stage 3 and 4 of endometriosis. The odds ratio (OR) was estimated using IVW method. The horizontal bars represent 95% confidence intervals (CI).

*Candidatus Soleaferrea*, *Eubacterium brachy* group, *Family_XIII AD3011* group, Ruminococcaceae *NK4A214* group, Ruminococcaceae *UCG002*, and *Sutterella* were associated with the risk of stage 1–2 of endometriosis (Supplementary Material S1; Supplementary Table S8) (Figure 3A). *Candidatus Soleaferrea*, Ruminococcaceae *NK4A214* group, and *Sutterella* showed significant causal associations with decreased risk of endometriosis in stage 1–2. The OR (95%CI) of *Candidatus Soleaferrea* in inverse variance weighted was 0.84 (0.72–0.98) (*p* = 0.029). The inverse variance weighted estimate of the Ruminococcaceae *NK4A214* group and *Sutterella* showed their protective effects on endometriosis in stages 1–2. The ORs (95%CI) were 0.77(0.61–0.97), 0.77(0.60–1.00), respectively. The inverse variance weighted estimate suggests that the *Eubacterium brach* group (OR = 1.16, 95%CI: 1.03–1.31, *p* = 0.015), *Family_XIII AD3011* group (OR = 1.26, 95%CI: 1.02–1.55, *p* = 0.032), and Ruminococcaceae *UCG002* (OR = 1.21, 95%CI: 1.02–1.43, *p* = 0.025) were risk factors on endometriosis in stages 1–2. We did not observe significant horizontal pleiotropy and heterogeneity on these gut microbiotas. There is no significant causal association between other gut microbiota and endometriosis in stages 1–2. (Supplementary Material S1; Supplementary Table S8).

### The casual association between genus of gut microbiota and moderate to severe endometriosis

We explored the causal effects of genus level of gut microbiota on endometriosis in ASRM stages 3–4. *Bacteroides*, *Holdemania*, and Lachnospiraceae NK4A136 group were protective factors in developing endometriosis in stages 3–4. The ORs (95%CI) were 0.79(0.62–0.99), 0.88(0.77–1.00), and 0.82 (0.71–0.95), respectively. We found no obvious horizontal pleiotropy and heterogeneity. The inverse variance weighted estimates did not support the causal associations of other gut microbiota on endometriosis in stages 3–4. (Supplementary Material S1; Supplementary Table S10) (Figure 3B).

## Discussion

Studies indicated that changes in the composition of gut microbiota may affect the development and progression of endometriosis. Comprehending these links could assist in identifying potential biomarkers for swift diagnosis and in developing personalized treatment approaches for patients with endometriosis. Furthermore, understanding the associations between gut microbiota and endometriosis might lead to the development of innovative treatment methods and improve clinical outcomes for individuals affected by this condition.

Recently, Muraoka et al. found that *Fusobacterium* infection activates the transforming growth factor-β (TNF-β) signal, promoting the progression of endometriosis. Antibiotic treatment effectively prevented the disease’s progression in a mouse model (Muraoka et al., 2023). Lipopolysaccharide (LPS) derived from gut microbiota can activate Toll-like receptor 4 (TLR4) and induce an inflammatory response, promoting the growth of endometriotic lesions. The production of TNF-α and IL-8, triggered by LPS activating TLR4, is crucial for endometrial tissue adhesion and angiogenesis (Nothnick, 2001). Meanwhile, Iba et al. found that treating endometrial stromal cells (ESCs) obtained from ovarian endometriosis with an NF-kB inhibitor resulted in reduced production of TNF-α and IL-8 and decreased proliferation (Iba et al., 2004). Khan et al. compared peritoneal fluid and menstrual fluid samples in endometriosis patients and controls, showing higher levels of LPS in the menstrual fluid of individuals with endometriosis (Khan et al., 2010). The presence of LPS may contribute to TLR4-mediated growth of endometriosis.

The gut microbiota can impact the structure and function of the intestinal epithelium and has been linked to various diseases, including hypertension, Parkinson’s disease, and autoimmune diseases (Sampson et al., 2016; Li et al., 2017; Janney et al., 2020; Mu et al., 2020; Christovich and Luo, 2022). The precise mechanism by which gut microbiota affects endometriosis remains uncertain. In this study, gut microbiota had causal effects on endometriosis, specifically *Holdemania* and Ruminococcaceae *UCG002*, which are associated with different stages of endometriosis.

Certain bacterial families, including *Burkholderiales*, Oxalobacteraceae, Porphyromonadaceae, and *Desulfovibrio*, may play a role in the development of endometriosis through the LPS-TLR4 pathway. They can produce LPS which is an essential component of the outer membrane. LPS activates immune cells or immune cell receptors, causing systemic inflammation responses and contributing to the development of endometriosis (Iba et al., 2004; Clemente et al., 2018; Gao et al., 2023).

LPS can activate the PD-L1 pathway, promote overexpression of PD-1 and PD-L1, and suppress T cell activation and proliferation. On the other hand, the *Clostridialesvadin BB60 group* lead to decreased PD-L1 levels, which can, in turn, suppress the growth of ectopic endometrial tissue in the pelvic cavity (Huang et al., 2023). As a result, the *Clostridialesvadin BB60 group* may reduce the incidence of endometriosis or alleviate its symptoms. The ectopic endometrial tissue can lead to inflammation and immune dysregulation, which can in turn affect the microbiome composition.

Xue Q et al. found that the abundance of *Haemophilus* increased significantly in patients with acute exacerbation of chronic obstructive pulmonary disease (COPD) (Xue et al., 2023). AECOPD is characterized by *Haemophilus* enrichment and a high level of TNF-α. *Haemophilus* is a respiratory pathogen specific to neutrophils (Sethi and Murphy, 2001). Neutrophils both promote tumor growth and inhibit tumor progression (Gungabeesoon et al., 2023). It exhibits anti-tumorigenic characteristics in the state of acute inflammation (Hirschhorn et al., 2023). Surprisingly, in this study, *Haemophilus* showed a protective effect in endometriosis. We hypothesize that endometriosis leads to inflammation in the body, and *Haemophilus,* through neutrophils, restricts the growth of ectopic endometrial tissue.

*Holdemania and* Ruminococcaceae *UCG002* are involved in gut butyrate production (Sampson et al., 2016). Butyrate plays a vital role in maintaining intestinal homeostasis and anti-inflammation. It can regulate the interaction between dendritic cells and DC-T cells and promote Treg T cell differentiation, thereby maintaining immune balance. This is achieved by HDACi suppressing the expression of NF-κB and inducing anti-inflammatory gene transcription to activate dendritic cells (Park et al., 2015).

We further investigated the causal link between the genus of gut microbiota and the different stages of endometriosis, revealing that *Holdemania* acted as a protective factor against stage 3–4 endometriosis. Conversely, higher levels of Ruminococcaceae *UCG002* increased the risk of stage 1–2 endometriosis. These findings were consistent with previous analyses and suggested that *Holdemania* and Ruminococcaceae *UCG002* may play essential roles in developing endometriosis. As mentioned, *Holdemania* and Ruminococcaceae *UCG002* participated in gut butyrate production (Gomez-Arango et al., 2018). These findings further indicated that gut microbiota worked in the occurrence and development of endometriosis. Notably, Ruminococcaceae *UCG002* is positively correlated with Treg cells (Zhi et al., 2022), and its abundance is closely related to the levels of pro-inflammatory cytokines. Exploring the detailed mechanisms by which *Holdemania* and Ruminococcaceae *UCG002* influence the progression of endometriosis should be focus on future research. The results are different between endometriosis states 1 and 2 and stages 3 and 4. We thought the lifestyle and treatment methods of patients with different stages of endometriosis may lead to changes in the composition of intestinal flora. Recent research has shown that lifestyle, diet, and other factors can affect gut microbiota composition. A comparison of the Hadza hunter and population in Nepal and California has revealed the impact of these factors (Carter et al., 2023).

In conclusion, these risk factors in gut microbiota mainly affect endometriosis through the LPS-TRL4 pathway. They induce local or systemic inflammation via releasing LPS, the concentrations of inflammatory mediators, cytokines, and chemokines are increased, and TRL4 is activated. This process promotes ectopic endometrial tissue angiogenesis and, thus, colonization. As for protective factors, they can reduce PD-1L levels, prevent angiogenesis, and inhibit the growth of ectopic endometrial tissue. Our study offers a new approach to treating endometriosis, reducing financial burden and relieving pain compared to conventional treatments. This benefits both patients and advances understanding of the condition.

There are several strengths in this study. Firstly, our study examines the relationship between gut microbiota and endometriosis at the genetic level, which reduces confounding bias and reverse causality. This study provides a new perspective for exploring the pathogenesis of endometriosis. Secondly, we have ensured two-sample Mendelian randomization (MR) analyses by selecting separate samples for exposure and outcome data. Thirdly, we have conducted multiple supplementary analyses, including heterogeneity, pleiotropy, leave-one-out sensitivity analyses, and MR-PRESSO, to ensure the robustness of our results.

Of course, several limitations also need to be acknowledged. Firstly, there are no data available at the species level. We cannot explicitly identify which bacteria affect endometriosis. Secondly, the summary statistic of gut microbiota includes multiple ancestries, which may introduce interference from population stratification in our results. Thirdly, the GWAS summary statistic for gut microbiota was not restricted to female participants. Although the exposure data have excluded the sex chromosomes, potential bias due to sex cannot be avoided entirely. In this study, some results were significant for only the IVW analysis. According to the supplementary analysis, there is horizontal pleiotropy or (and) heterogeneity. However, further MR-PRESSO analysis did not find any significant outliers. Therefore, it is necessary to verify our results through further clinical and basic research.

## Conclusion

This two-sample MR analysis revealed a causal association between certain gut microbiotas and endometriosis. These microbiotas primarily influence endometriosis through the LPS-TRL4 pathway and inflammatory factors. Moving forward, it is essential to consider the design of randomized controlled trials (RCTs) and fundamental studies to elucidate further the risk and protective factors associated with endometriosis and its underlying mechanisms.

## Data Availability

The original contributions presented in the study are included in the article/Supplementary Material, further inquiries can be directed to the corresponding author.

## Supplementary material

SUPPLEMENTARY FIGURE S1 Plots of leave-one-out for relationship between gut microbiota and endometriosis in FinnGen.

SUPPLEMENTARY FIGURE S2 Scatter plots for the causal effects of gut microbiota on endometriosis in FinnGen cohort.

SUPPLEMENTARY FIGURE S3 Plots of leave-one-out for relationship between gut microbiota and endometriosis in stage 1 and 2.

SUPPLEMENTARY FIGURE S4 Scatter plots for the causal effects of gut microbiota on endometriosis in stage 1 and 2.

SUPPLEMENTARY FIGURE S5 Plots of leave-one-out for relationship between gut microbiota and endometriosis in stage 3 and 4.

SUPPLEMENTARY FIGURE S6 Scatter plots for the causal effects of gut microbiota on endometriosis in stage 3 and 4.

SUPPLEMENTARY TABLE S1 Instrumental variables for genus of gut microbiota in this study.

SUPPLEMENTARY TABLE S2 MR analysis results between phylum of gut microbiota and endometriosis in FinnGen.

SUPPLEMENTARY TABLE S3 MR analysis results between class of gut microbiota and endometriosis in FinnGen.

SUPPLEMENTARY TABLE S4 MR analysis results between order of gut microbiota and endometriosis in FinnGen.

SUPPLEMENTARY TABLE S5 MR analysis results between family of gut microbiota and endometriosis in FinnGen.

SUPPLEMENTARY TABLE S6 MR analysis results between genus of gut microbiota and endometriosis in FinnGen.

SUPPLEMENTARY TABLE S7 MR-PRESSO results between genus of gut microbiota and endometriosis in FinnGen.

SUPPLEMENTARY TABLE S8 The results of MR analysis between gut microbiota and endometriosis in stage 1 and 2.

SUPPLEMENTARY TABLE S9 MR-PRESSO results between genus of gut microbiota and endometriosis in stage 1 and 2.

SUPPLEMENTARY TABLE S10 The results of MR analysis between gut microbiota and endometriosis in stage 3 and 4.

SUPPLEMENTARY TABLE S11 MR-PRESSO results between genus of gut microbiota and endometriosis in stage 3 and 4.
