# Supplementary figures and images for: Endometriosis is a disease of immune dysfunction, which could be linked to microbiota
Source: Front Genet. 2024 Jun 21;15:1386411. doi: 10.3389/fgene.2024.1386411 (PMC11227297; doi:10.3389/fgene.2024.1386411)

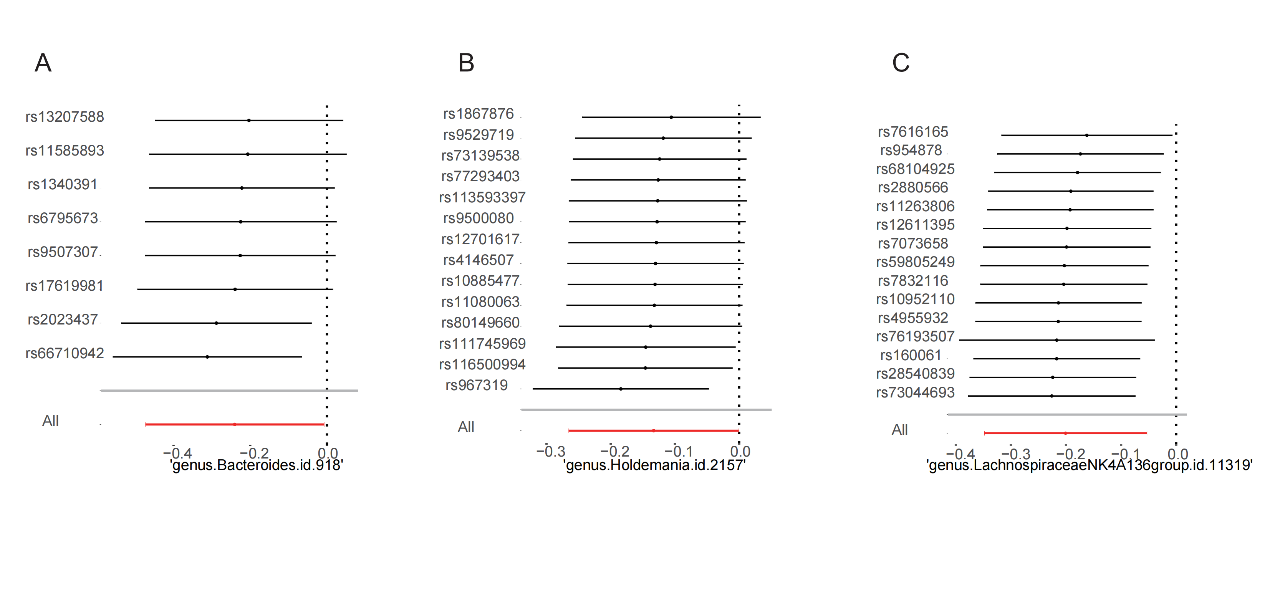

Supplement: Supplementary file 1 [file Image5.PNG]

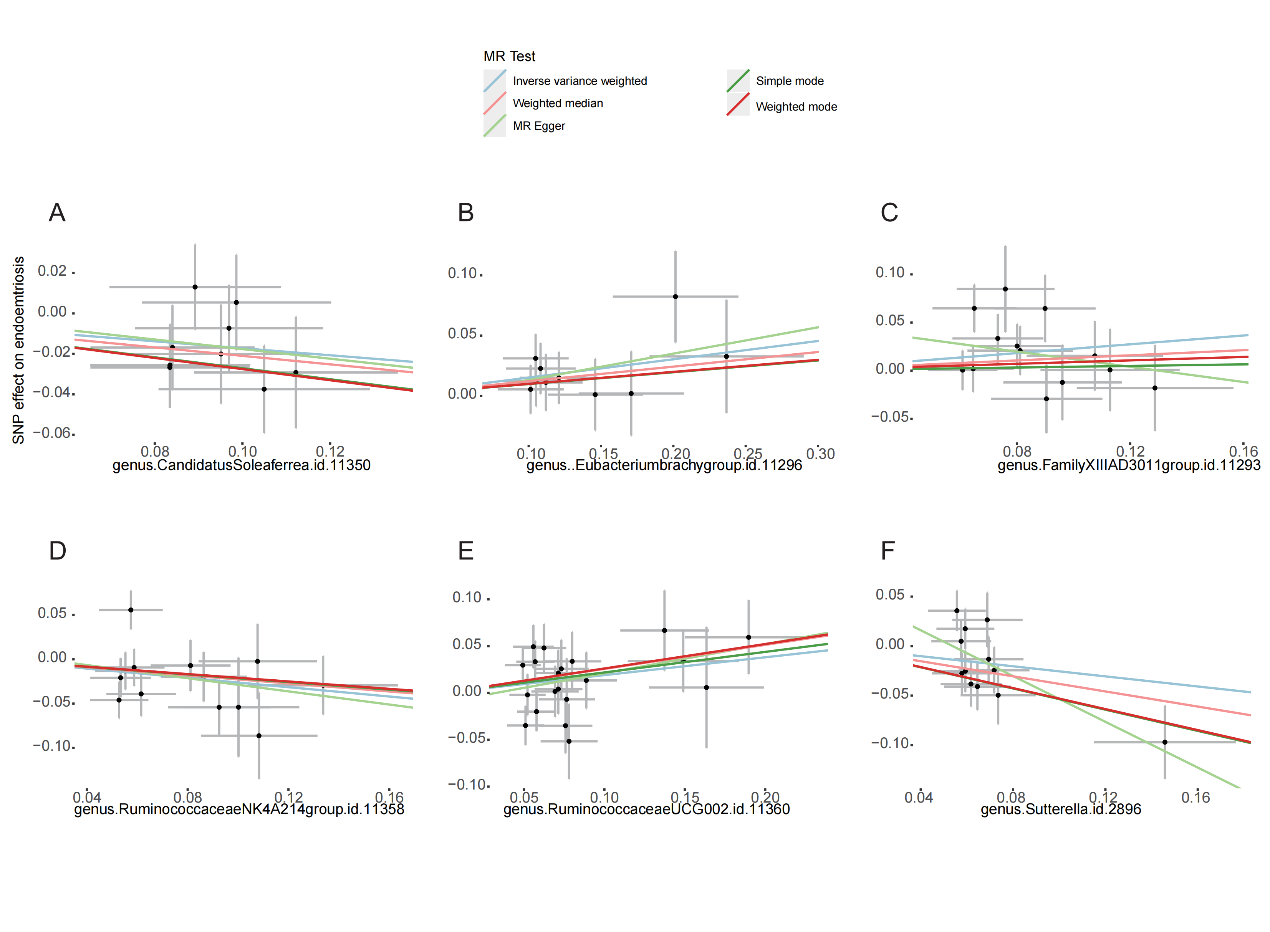

Supplement: Supplementary file 2 [file Image4.PNG]

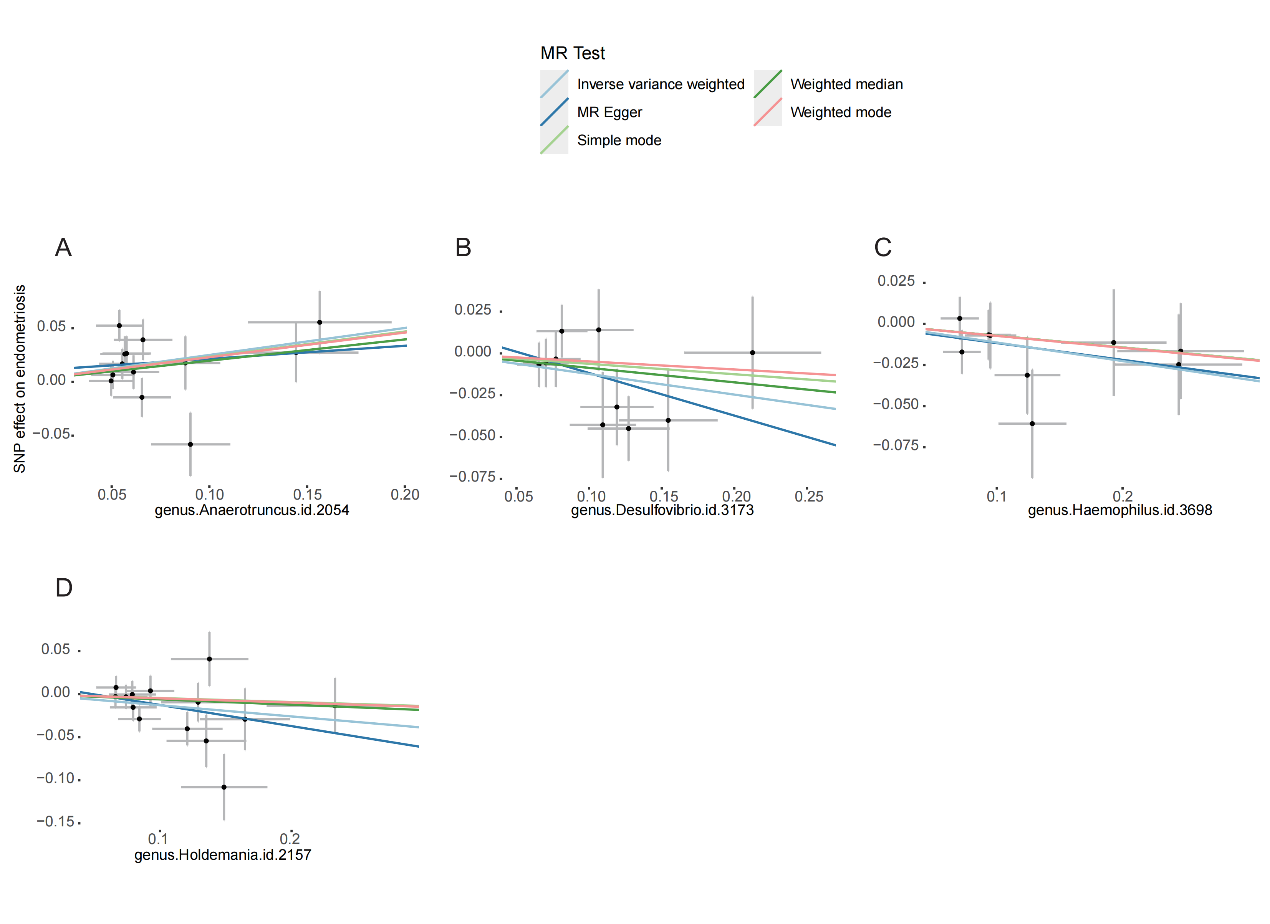

Supplement: Supplementary file 3 [file Image2.PNG]

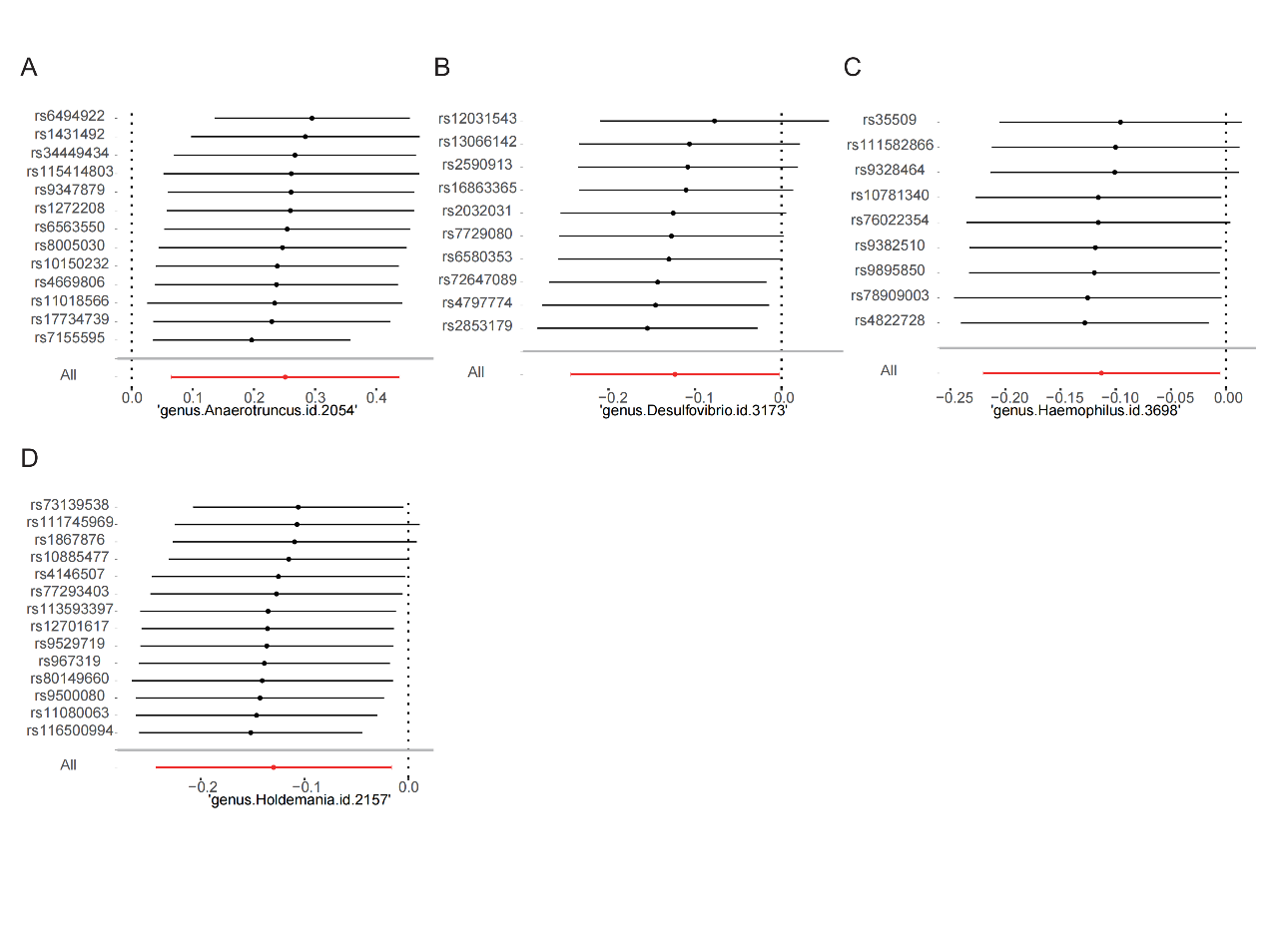

Supplement: Supplementary file 5 [file Image1.PNG]

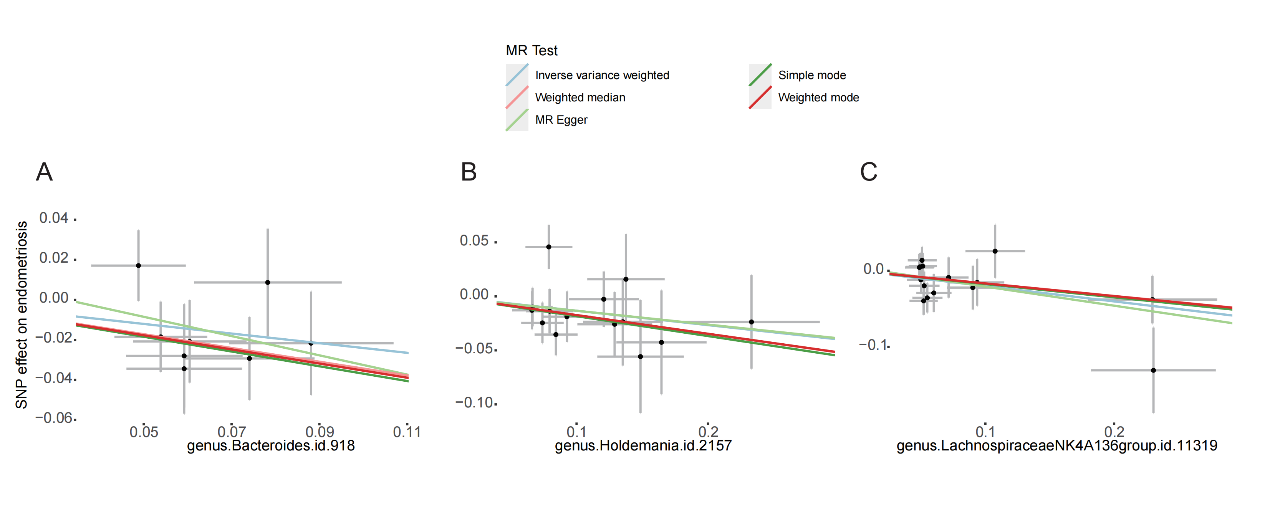

Supplement: Supplementary file 6 [file Image6.PNG]

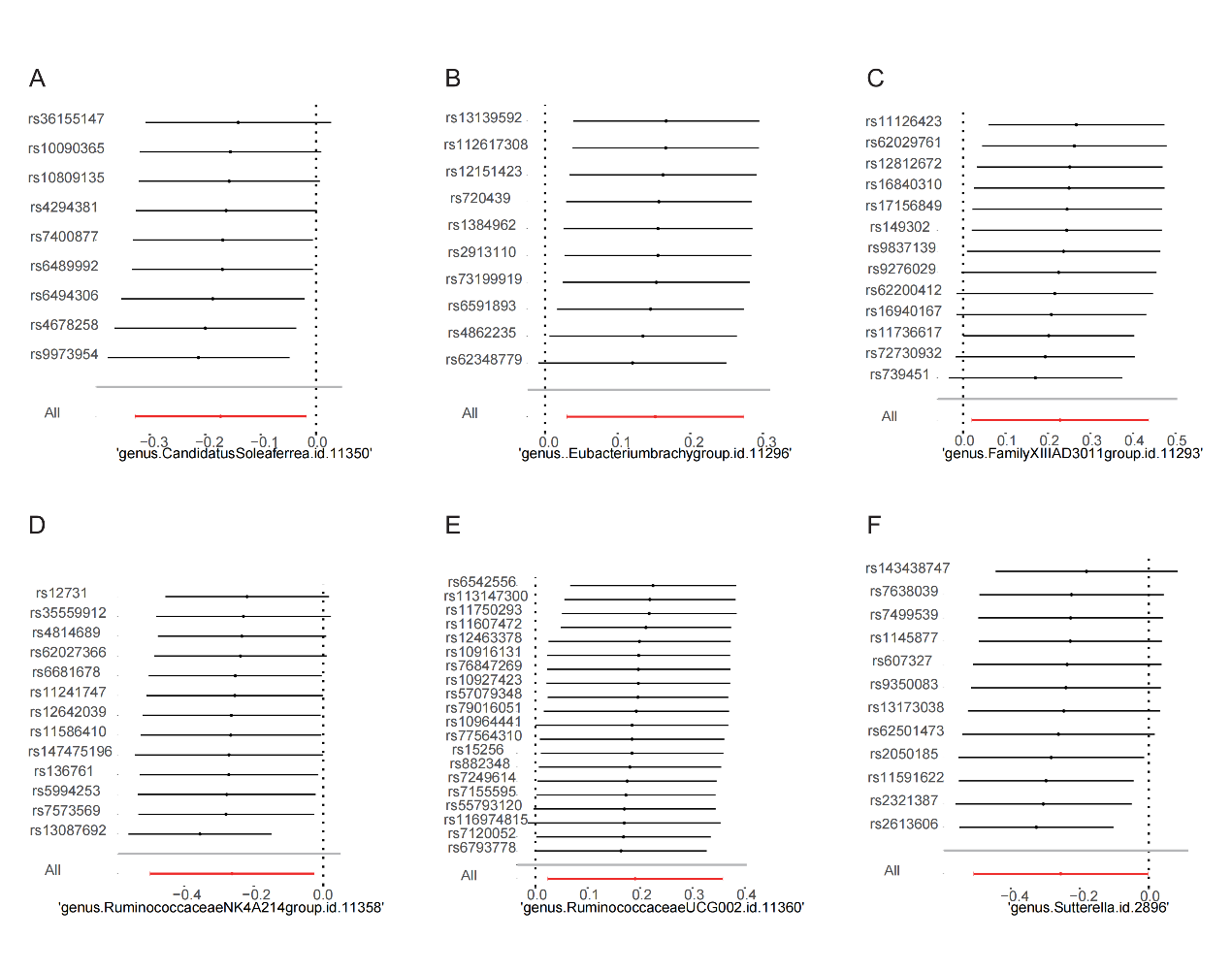

Supplement: Supplementary file 7 [file Image3.PNG]
